# Non-Antibody-Based Binders for the Enrichment of Proteins for Analysis by Mass Spectrometry

**DOI:** 10.3390/biom11121791

**Published:** 2021-11-30

**Authors:** Oladapo Olaleye, Natalia Govorukhina, Nico C. van de Merbel, Rainer Bischoff

**Affiliations:** 1 Department of Analytical Biochemistry, Groningen Research Institute of Pharmacy, University of Groningen, A Deusinglaan 1, 9713 AV Groningen, The Netherlands; O.O.Olaleye@rug.nl (O.O.); N.Govorukhina@rug.nl (N.G.); MerbelNicovande@prahs.com (N.C.v.d.M.); 2 Bioanalytical Laboratory, ICON, Amerikaweg 18, 9407 TK Assen, The Netherlands

**Keywords:** mass spectrometry, affimer, antibody, phage display, protein analysis

## Abstract

There is often a need to isolate proteins from body fluids, such as plasma or serum, prior to further analysis with (targeted) mass spectrometry. Although immunoglobulin or antibody-based binders have been successful in this regard, they possess certain disadvantages, which stimulated the development and validation of alternative, non-antibody-based binders. These binders are based on different protein scaffolds and are often selected and optimized using phage or other display technologies. This review focuses on several non-antibody-based binders in the context of enriching proteins for subsequent liquid chromatography-mass spectrometry (LC-MS) analysis and compares them to antibodies. In addition, we give a brief introduction to approaches for the immobilization of binders. The combination of non-antibody-based binders and targeted mass spectrometry is promising in areas, like regulated bioanalysis of therapeutic proteins or the quantification of biomarkers. However, the rather limited commercial availability of these binders presents a bottleneck that needs to be addressed.

## 1. Introduction

The analysis of proteins from matrices, such as plasma or serum, is challenging due to their complexity [1], the large dynamic concentration range estimated to reach 12 orders of magnitude [2], and the fact that albumin, immunoglobulins, transferrin, haptoglobin, and lipoproteins make up more than 90% of the amount of blood proteins [3]. While Ligand Binding Assays (LBAs) make use of the high specificity of affinity binders to discriminate target proteins from a background, Liquid Chromatography-Mass Spectrometry (LC-MS) assays, for example, in the Selected Reaction Monitoring (SRM) mode, make use of the mass-to-charge (*m*/*z*) ratio of so-called signature peptides, their characteristic fragmentation patterns, as well as their retention times upon Reversed-Phase Liquid Chromatography (RPLC) to achieve the necessary selectivity. While LC-MS assays have notable advantages over LBAs in terms of precision and accuracy and their ability to quantify multiple proteins in one analysis, they often suffer from insufficient concentration sensitivity when compared to LBAs. To overcome this shortcoming, it is necessary to include an enrichment step prior to the actual LC-MS analysis to capture the target protein and to remove interfering plasma or serum proteins [4]. This is particularly critical when attempting to quantify proteins at the picomolar (ng/mL and below) level, a range where many biopharmaceuticals and biomarkers of interest are found.

**Figure 1:**
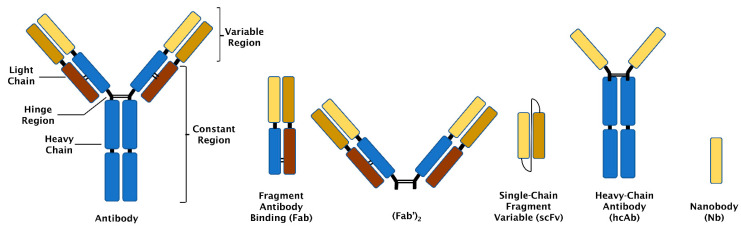
Schematic structures of different antibody-based binders.

**Figure 2:**
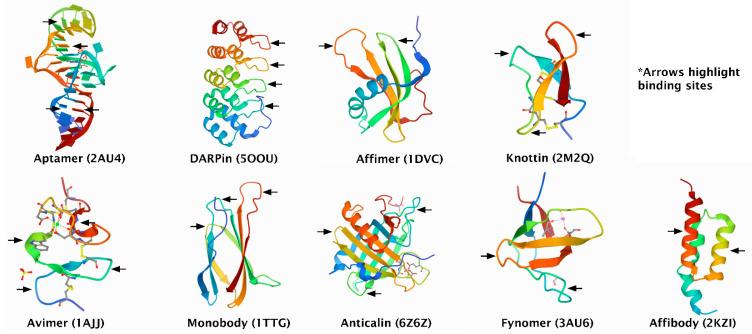
Crystal structures of non-antibody-based binders. Obtained from the Protein Data Bank, accession numbers in brackets (PDB, http://www.rcsb.org/ accessed on 3 October 2021).

Affinity enrichment requires binders that capture a given protein or a set of proteins with high specificity [5,6]. Binders may be antibody-based (Figure 1 and Table 1) or based on other protein scaffolds [7] (Table 2 and Figure 2). In addition, binders could also be transition metals, such as Zn^2+^, Cu^2+^, Ni^2+^, or Co^2+^, which target electron donor groups on certain amino acids [8]. This review will focus on non-antibody-based affinity binders and compare their performance with antibody-based binders using a number of selected examples.

## 2. Antibody-Based Binders

### 2.1. Antibodies

Antibodies are Y-shaped proteins (~150 kDa) containing two identical heavy and light chains linked together by disulphide bonds (Figure 1) [9]. Antibodies possess constant and variable regions. The variable regions contain the antigen-binding sites [46], while the constant regions are responsible for the effector functions of the antibody. The most currently used antibodies are monoclonal and can be produced via hybridoma technology after immunisation of a suitable animal or by recombinant DNA technology and subsequent expression in mammalian cell lines [10].

### 2.2. Fragment Antibody Binding (Fab)

Fab is an antibody fragment (~50 kDa) that contains one constant and one variable region each from the light and heavy chains [11]. Fabs are produced by limited proteolysis with papain [12], IdeS [13], or GingisKHAN™ [47], which cleave at or near the hinge region. The corresponding sequences may also be cloned and expressed in mammalian cell lines [12], yeast [11], or *Escherichia coli* [48]. Two Fab may be linked by an intramolecular disulphide bond (see Figure 1) to form (Fab’)_2_. Fab has one antigen-binding region, while (Fab’)_2_ has two binding sites. Fab and (Fab’)_2_ lack the Fc part of antibodies and thus cannot exert any of the effector functions [14].

### 2.3. Single Chain Fragment Variable (scFv)

An scFv consists of two variable fragments (~25 kDa) each from the light and heavy chains of an antibody joined by a peptide linker [15,16]. scFvs are produced by recombinant DNA technology and expression in yeast or bacterial cells [17].

### 2.4. Heavy Chain Antibodies (hcAbs)

These are antibodies (~75 kDa) first discovered in camels and which do not have light chains. Their antigen-binding sites are determined by one variable region of the heavy chain [18]. hcAbs are produced by hybridoma technology after immunisation of a suitable animal or recombinant DNA technology and subsequent expression in mammalian cells [19].

### 2.5. Nanobodies (Nbs)

A nanobody is defined by only one variable region of an hcAb and is the smallest antibody-based binder (~15 kDa). Nbs are produced by recombinant DNA technology and expression in bacteria, mammalian cell lines, or plants [49].

Although important achievements and successes have been realised with antibody-based binders as capture agents, certain challenges have been encountered. Antibodies have a complex and expensive production process [50,51] and have shown batch-to-batch variability [52,53]. Antibodies may also lose their ability to bind their target antigen during immobilisation (attachment to a surface) [54]. Fabs and scFvs generally show lower affinity to targets when compared to antibodies, and Fabs are harder to customise than scFvs for immobilisation purposes [20]. scFvs are easier and cheaper to produce [15] but are less stable due to aggregation [16,55,56]. hcAbs and Nbs are somewhat limited with respect to binding site engineering since their antigen-binding sites are determined by one variable region only [21]. These challenges have incited researchers to develop alternative binders that retain the advantages of antibody-based binders, such as specificity and high affinity, while avoiding some of the disadvantages mentioned above.

## 3. Non-Antibody-Based Binders

Table 2 gives an overview of alternative binders that are not based on or derived from antibodies.

### 3.1. Aptamers

Aptamers are based on oligonucleotide or peptide structures [22] with specificity and affinity comparable to antibodies [20]. Oligonucleotide aptamers have a size range of about 5–30 kDa and are target-selected via an in-vitro method termed Systematic Evolution of Ligands by Exponential Enrichment (SELEX) [57]. SELEX is a combinatorial selection method that identifies oligonucleotide aptamers with significant affinity and binding specificity to ligands, such as proteins [23,58]. Peptide aptamers are based on a randomised set of peptide sequences engineered into a stable protein scaffold [24]. The optimal peptide sequence is selected from a peptide aptamer library intracellularly using yeast or mammalian cells or extracellularly by phage display technology. For the intracellular process, the peptide aptamer is introduced into yeast cells and exposed to a target via a two-hybrid system. Selection is achieved by interaction traps so that the ability to grow in certain media depends on the interaction of a suitable peptide with the target [25]. The selected peptide sequence(s) is then inserted into a protein scaffold, such as bacterial thioredoxin, and expressed in *Escherichia coli* or yeast cells [59]. A disadvantage of oligonucleotide aptamers is that they can be degraded by nucleases [26], but certain strategies, such as phosphodiester backbone or sugar ring modifications, allow prevention of this [27].

### 3.2. DARPins (Designed Ankyrin Repeat Proteins)

DARPins are proteins with a size of about 14–18 kDa [28] comprised of ankyrin repeats, which assemble to form an overlapping binding surface [60]. The structure of each ankyrin repeat is defined by a beta-turn along with two antiparallel alpha-helices [29]. An interesting characteristic of ankyrin repeats is that their original biological function is the selective binding to target proteins [30]. Selective DARPins are isolated using phage or ribosome displays [31,32] and can be expressed at considerable levels in *Escherichia coli* [29]. DARPins are not susceptible to aggregation even at high concentrations [29]. The ability of DARPins to bind certain targets may be limited by their concave binding surface and restrictions to fully randomizing all amino acids in their binding sites [28].

### 3.3. Affimers

Affimers are based on the human protease inhibitor stefin A or phytocystatin protein scaffolds and are about 12–14 kDa in size [33,61]. They contain two variable binding loops, and their structure consists of four beta-sheets and one alpha-helix. The binding loops comprise about nine amino acids that can be modified at random using phage display technology to bind different targets [62]. Affimers can be expressed at high yields in *Escherichia coli* [61]. Affimers have been reported to show some form of aggregation [63,64].

### 3.4. Knottins

Knottins are small-sized proteins with at least three disulphide bridges [34] that are based on the inhibitor cysteine knot (ICK) scaffold [35]. They are approximately 4 kDa in size [36]. Knottins are characterised by a structure in which one disulphide bridge passes through a cyclic framework created by the peptide backbone and the two other disulphide bridges. This knot-like structure brings about high thermal and proteolytic stability. Knottins can be produced via chemical synthesis or recombinant expression in yeast cells after selection by yeast display [65]. Yeast display involves the expression of recombinant proteins as part of the cell wall of yeast for the selection and engineering of affinity binders. The binders are fused to a cell surface receptor, such as a-agglutinin, to enable exposure to a target and the inclusion of fluorescent tags enables sorting and selection of suitable binders by flow cytometry [66]. An important advantage of yeast display is that it can provide affinity binders that possess post-translational modifications [37]. Knottins contain loop regions of variable lengths that have been reported to accept non-natural amino acids and can be engineered for binding to different targets. Non-natural amino acids can be introduced via chemical synthesis into these loops [67].

### 3.5. Avimers

Avimers are approximately 4 kDa in size [38], based on the conserved A-domain of different cell surface receptors [36]. Avimers are produced by a selection of distinct binding sites, thereby providing a protein that is capable of binding different sites on the same target or even binding to different targets simultaneously [39]. Avimers can be expressed at considerable amounts without perceptible inclusion body formation in *Escherichia coli* after the selection of optimal binders by phage display [38]. The fact that avimers require calcium for stability may be disadvantageous. This is because chelating agents, such as EDTA (often used in preparing blood plasma), may prevent binding [7].

### 3.6. Monobodies

Monobodies are binding proteins generated from the human fibronectin type III domain (FN3) [40] with an approximate size of 10 kDa [41]. Monobodies can be selected by phage or yeast display and efficiently expressed in *Escherichia coli* [68].

### 3.7. Anticalins

Anticalins are protein binding fragments of 20 kDa [42] that are based on the structure of lipocalins. Lipocalins are a group of secreted proteins that transport hydrophobic compounds [36]. The interest in lipocalins as a basis for binders is due to structural similarity between their four binding loops and that of the six complementary determining regions (CDRs) in antibodies [43]. Anticalins can be expressed in *Escherichia coli* after selection with phage display [44].

### 3.8. Fynomers

Fynomers are binding proteins obtained from the human tyrosine kinase Src Homology 3 (SH3) domain [45]. Fynomers are about 7 kDa in size and are selected via phage display followed by an expression in *Escherichia coli* [69].

### 3.9. Affibodies

Affibodies are binding proteins based on the three-helix bundle Z domain scaffold. The Z domain is derived from the B domain of *Staphylococcus aureus* protein A [70]. Affibodies are about 7 kDa in size and can be produced by chemical synthesis as well as by expression in *Escherichia coli* after selection with phage display [36,69].

## 4. Phage Display

**Figure 3:**
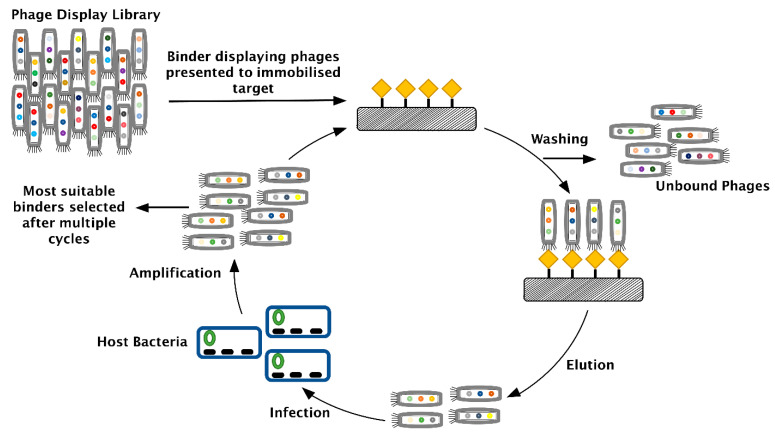
The process of biopanning as part of phage display for selecting highly specific binders against a given target.

Since many of the non-antibody-based binders are selected through phage display or a similar display technique, it is appropriate to introduce this approach briefly. Phage display is a molecular biology technique in which large, highly variable sequence libraries are presented as peptides or proteins on filamentous phage surfaces to enable the selection of binders with specificity and high affinity to almost any target, provided the target protein is available in sufficient purity (quality) and amount [71]. To select a binder for a target using phage display, a process referred to as biopanning is performed. Biopanning involves the exposure of a phage library to a target followed by multiple steps of washing, elution, and amplification to obtain the most suitable binders for that target [72]. Figure 3 shows the steps involved in biopanning.

For a successful selection of binders using this approach, it is critical to provide a well-characterized target protein and to consider a meaningful counter screening approach to avoid selecting binders for interfering matrix proteins. This is not always easy, especially when the target protein is not available in pure form or cannot be produced in a form that mimics the target protein (e.g., an endogenous target protein may have different post-translational modifications than its recombinant counterpart used for screening). Even with a well-designed screening procedure, non-antibody-based binders are not entirely specific, just like their antibody-based counterparts. However, the combined selectivity of the binder and the LC-MS procedure often result in bioanalytical methods that work successfully for the quantification of proteins in complex biological matrices, such as serum or plasma.

## 5. Immobilisation Approaches

The attachment of a binder to a surface to restrict or limit its movement is referred to as immobilization [73]. By far, most enrichment procedures are based on immobilizing the binder to a surface. It is important to consider that immobilizing a binder should not interfere with binding to the target protein. The effectiveness of a binder after immobilization depends on its orientation as well as its accessibility (e.g., when working with porous surfaces). A binder can be described as having a proper orientation if immobilisation does not obstruct its binding regions [74], as this may result in reduced recovery of the target protein [75]. Immobilization approaches may be discriminated on whether they are based on physical adsorption or covalent bonds as well as on whether they result in random orientation of the immobilized binder or a defined orientation. Often, it is favourable to immobilize a binder covalently in a defined orientation (at a defined site), but this is not always possible.

### 5.1. Physical Adsorption

This makes use of the ability of proteins to adsorb to surfaces through intermolecular forces, such as electrostatic, hydrogen bonding, dipole-dipole, or hydrophobic interactions [73]. Although physical adsorption is the easiest method, orientation cannot be controlled, and certain proteins, such as antibodies [75], may denature and lose their binding capacity. Since there is often a large excess of immobilized binder over target protein, some loss of binding capacity may be acceptable in favour of having an easy-to-use method. However, this must be carefully controlled to avoid problems with reproducibility, notably when quantitative results are needed. Another risk of immobilizing binders by physical adsorption is that they may be (partially) washed off the surface during the enrichment procedure [73,75]. This is why physical adsorption is not widely used in combination with (LC)-MS assays.

### 5.2. Covalent Immobilisation

The formation of covalent bonds between functional groups of exposed side chains in proteins and corresponding surface-bound functional groups plays an important role in hyphenated affinity enrichment (LC)-MS assays. There are a number of functional groups on proteins that may be used for immobilization, such as -COOH, -SH, and NH_2_ groups, as well as activated groups on surfaces or activating agents (also called coupling reagents). The use of promiscuous functional groups on a binder for immobilisation is random and may lead to improper orientation. However, this is avoided and the immobilisation made selective and site-specific if a uniquely reactive functional group is localised at a predetermined site on the binder [73]. Amino acids containing such functional groups may be introduced by chemical synthesis or genetic code reprogramming [76]. It is outside the scope of this review to summarize the many possibilities of covalent immobilization of proteins. We refer the reader to reviews on this topic [77,78,79].

### 5.3. Affinity Immobilisation

This involves the use of the affinity interactions between a binder and a corresponding surface. Binders may have to be modified to be suitable for immobilization. The interaction of biotin with streptavidin, of a His-tag with Ni^2+^ ions, or protein A/G with the Fc region of antibodies are commonly used for non-covalent, affinity-based immobilization. Since affinity interactions are often strong and specific, the risk of washing the binder off the surface is low. Affinity immobilisation is gentle compared to other methods, and it also provides a possibility to reuse a surface or support as binders can be detached and fresh binders reattached [73].

In this context, it is important to consider how to elute (recover) the bound target protein from the immobilized binder. This is often done under denaturing conditions, such as at a low pH. Other options are to directly digest the captured protein(s) on the surface to obtain the required signature peptides for LC-MS analysis. This approach, while often more quantitative, has the disadvantage of producing peptides from the immobilized binder, which is often in excess of the captured target protein. This is often not a problem for targeted LC-MS assays since the signature peptides of interest can be discriminated from other peptides based on their *m*/*z* values, their fragmentation patterns, and their retention times. When gentler elution conditions are required, for example, to perform functional studies, reversible covalent chemistry may provide options to immobilize a binder covalently but to recover it together with the target protein(s) based on a specific chemical reaction [80].

## 6. Non-Antibody-Based Affinity Enrichment of Proteins in Combination with Mass Spectrometry

While antibodies are still most widely used for protein enrichment prior to (LC)-MS analysis, there are a number of examples where non-antibody-based binders were employed and sometimes also compared with antibodies. Affimers were compared to antibodies for the enrichment of the recombinant forms of two proteins, interleukin 37 (IL-37) and proinsulin, from plasma in a study by Tans et al. [81]. IL-37 is a cytokine with anti-inflammatory properties, while proinsulin is the precursor of insulin, which is important for controlling blood glucose levels. Both sets of binders were biotinylated and immobilised on magnetic streptavidin beads for the enrichment process, after which digestion was performed, followed by analysis with mass spectrometry. Western blot analysis showed that the affimers were able to enrich proinsulin more efficiently than a commercially available monoclonal antibody, which was confirmed by shotgun proteomics.

**Figure 4:**
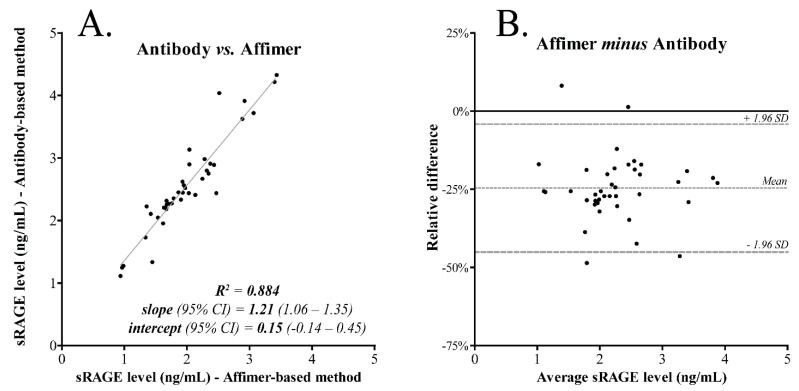
Comparison between an affimer-based sRAGE LC-MS assay and a previously developed antibody-based LC-MS method for sRAGE analysis in 40 serum samples. (**A**) correlation between results by linear regression and (**B**) bias by Bland−Altman plot (reproduced with permission from [82]).

Klont et al. [82] used affimers in a fully validated assay to quantify the soluble receptor of advanced glycation end-products (sRAGE) in serum as a potential biomarker for emphysema development in Chronic Obstructive Pulmonary Disease (COPD). The affimers were immobilised on microtiter plates, and the captured sRAGE was analysed by LC-MS in the SRM mode after digestion with trypsin. The results were compared with a validated method that used immobilized antibodies on microtiter plates followed by the same SRM assay (Figure 4). Data showed that there was a good correlation between the two assays but that the antibody-based method gave about 25% higher sRAGE concentrations [83]. The reason for this discrepancy remained unexplained.

Radko et al. [84] evaluated the enrichment of recombinant SMAD4 protein from plasma using oligonucleotide-based aptamers. SMAD4 is a tumour suppressor and controls the growth of epithelial cells by mediating the TGF-β signalling pathway [85]. SMAD4 has been linked to pancreatic and colorectal cancers [86]. The aptamers were biotinylated and immobilized on magnetic streptavidin beads. The resulting digests were analysed with LC-MS in SRM mode. Two signature peptides were used for the quantification of SMAD4. It was reported that enrichment of SMAD4 prior to LC-MS analysis resulted in a gain in concentration sensitivity of 1.5 orders of magnitude as compared to direct, in-plasma digestion.

Using a panel of aptamers, Ngo et al. [87] attempted to define biomarkers involved in cardiovascular disease. After being coupled to magnetic beads, the aptamers were used for the pull-down of selected proteins spiked in plasma. Successful enrichment of the proteins was confirmed by a tryptic digest and subsequent analysis with SRM.

In another study, the ability of an aptamer to capture recombinant PCSK9 from plasma was compared to an antibody-based approach by Gupta et al. [88]. PCSK9 is involved in blood cholesterol regulation [89]. Both affinity binders were coupled to magnetic beads, and the resulting samples were digested and analysed by LC-MS in the SRM mode. The aptamer produced comparable results to the antibody.

**Figure 5:**
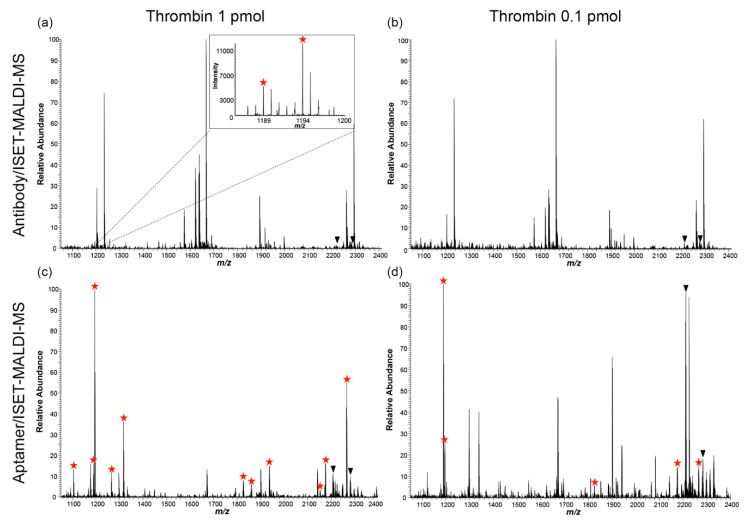
MALDI-MS spectrum comparison between the antibody-based (**a**,**b**) and affimer-based (**c**,**d**) affinity enrichment methods. Thrombin-derived peptides are indicated with red stars. More peptides are observed at the 1 pmol level with the aptamer-based approach (**c**) when compared to the antibody-based approach (**a**). Thrombin can only be detected after aptamer-based enrichment at the 0.1 pmol level (**d**), while only background peptides are visible after antibody-based enrichment (reproduced with permission from [90]).

Lee et al. [90], using matrix-assisted laser desorption/ionization (MALDI) MS, studied the affinity enrichment of thrombin by an aptamer and an antibody while immobilised on beads. MALDI MS analysis of the tryptic digest resulting from the aptamer-based enrichment resulted in more thrombin-derived peptides and a reduced background when compared to the antibody-based method. Additionally, thrombin was detected at a lower concentration with the aptamer-based approach, while it was undetectable at the same concentration with the antibody-based enrichment approach (Figure 5).

## 7. Conclusions and Perspectives

LC-MS has made its entry in the field of protein bioanalysis and has been shown to outperform many of the widely used ligand binding assays (LBAs) in terms of accuracy and precision. This is particularly important in the field of regulated bioanalysis, for example, in the pharmaceutical industry, in governmental analytical laboratories, as well as in hospitals. However, LC-MS-based methods often suffer from a lack of sensitivity when compared to LBAs. Including an affinity enrichment step prior to the actual LC-MS assay overcomes some of these limitations although it is fair to say that modern formats of LBAs, such as those based on single-molecule analysis, still outperform even hybrid LC-MS assays in terms of sensitivity. The biggest advantage of hybrid LC-MS assays is likely that the mass spectrometric readout provides concrete chemical information about the analyte in contrast to LBAs, which generate indirect (e.g., chemoluminescent) readouts that may also derive from non-specifically bound interfering proteins. It is thus likely that hybrid affinity enrichment LC-MS assays will be more extensively used in areas where accuracy, precision, and the avoidance of false positives are critical.

Significant progress has been made in the development of non-antibody-based binders over the past 20 years, and their ability to bind target proteins with high affinity and specificity has been demonstrated [91,92]. Unlike most antibody-based binders, they are easier and cheaper to produce in bacterial systems or via chemical synthesis. Non-antibody-based binders are often more stable, which suggests that they can be used across a wider range of conditions and that they may even be reusable if so desired. Selective or site-specific immobilisation is less challenging to achieve with non-antibody-based binders due to their smaller size and the possibility to introduce defined functional groups in a site-specific manner.

Taken together, this makes non-antibody-based binders and LC-MS an ideal combination for future bioanalytical assay development. However, the wide commercial availability of antibodies and their long track record in the bioanalysis of proteins mean that they are still the most widely used affinity reagents today. To change this situation, it is necessary that non-antibody-based binders become available to a larger community outside the often specialist laboratories and companies that developed them. The limited commercial availability of many non-antibody-based binders, for reasons of confidentiality or intellectual property protection, hampers their widespread use. We hope that this situation will change in the future so that all researchers can benefit from the unique properties of non-antibody-based binders.

## Figures and Tables

**Table 1:** Antibody-Based Binders.

Antibody-Based Binder	Size	Production	Refs
Antibody	~150 kDa	Hybridoma or recombinant DNA technology and mammalian cell expression	[8,9]
Fragment Antibody Binding (Fab)	~50 kDa	Proteolysis (e.g., with papain, IdeS, or GingisKHAN™) or recombinant DNA technology and mammalian, yeast, or bacterial cell expression	[10,11,12,13]
Single-Chain Fragment Variable (scFv)	~25 kDa	Recombinant DNA technology and yeast or bacterial cell expression	[14,15,16]
Heavy Chain Antibodies	~75 kDa	Hybridoma or recombinant DNA technology and mammalian cell expression	[17,18]
Nanobodies	~15 kDa	Recombinant DNA technology and plant, mammalian, or bacterial cell expression	[19]

**Table 2:** Non-antibody-based binders and their characteristics.

Non-Antibody-Based Binder	Scaffold	Size	Production	Refs
Aptamers	Oligonucleotide/Protein scaffolds	5–30 kDa	Chemical synthesis as part of the Systematic Evolution of Ligands by Exponential Enrichment (SELEX) procedure/Phage display and bacterial expression	[20,21,22,23,24,25]
DARPins	Ankyrin repeats	14–18 kDa	Phage or ribosome display and bacterial expression	[26,27,28,29,30]
Affimers	Human stefin A or phytocystatin	12–14 kDa	Phage display and bacterial expression	[31,32,33]
Knottins	Inhibitor cysteine knot	~4 kDa	Chemical synthesis or yeast display and yeast expression	[34,35,36]
Avimers	A-domain region of cells	~4 kDa	Phage display and bacterial expression	[35,37]
Monobodies	Human fibronectin type III domain	~10 kDa	Phage or yeast display and bacterial expression	[38,39,40]
Anticalins	Lipocalins	~20 kDa	Phage display and bacterial expression	[41,42]
Fynomers	Human tyrosine kinase Src Homology 3 domain	~7 kDa	Phage display and bacterial expression	[43,44]
Affibodies	*S. aureus* Protein A	~7 kDa	Phage display and bacterial expression	[36,44,45]
